# Temporal patterns of blood flow and nitric oxide synthase expression affect macrophage accumulation and proliferation during collateral growth

**DOI:** 10.1186/2040-2384-2-18

**Published:** 2010-09-16

**Authors:** Hendrik B Sager, Ralf Middendorff, Kim Rauche, Joachim Weil, Wolfgang Lieb, Heribert Schunkert, Wulf D Ito

**Affiliations:** 1 Medizinische Klinik II, Universität zu Lübeck Ratzeburger Allee 160, D-23538 Lübeck, Germany; 2 Institut für Anatomie und Zellbiologie, Justus-Liebig Universität Giessen Aulweg 123, D-35385 Giessen, Germany; 3 Herz- und Gefäßzentrum Oberallgäu-Kempten, Klinik Immenstadt Im Stillen 2, D-87509 Immenstadt, Germany

## Abstract

**Background:**

The involvement of collateral blood flow/fluid shear stress, nitric oxide (NO), and macrophages during collateral growth (arteriogenesis) is established, but their interplay remains paradoxical.

**Methods:**

In order to further elucidate the "fluid shear stress/NO/macrophage" paradox, we investigated the time course of collateral blood flow (using a Doppler flow probe) and NOS expression (immunohistochemistry, Western blot) in growing rat collateral vessels after femoral artery occlusion and their impact on macrophage recruitment and collateral proliferation (immunohistochemistry, angiographies).

**Results:**

(values are given as mean ± standard error of mean) Early after occlusion, collateral blood flow was significantly reduced (pre- 90.0 ± 4.5 vs. post-occlusion 62.5 ± 5.9 μl/min; *p *< 0.01), and local inducible NOS (iNOS) and endothelial NOS (eNOS) expression were down-regulated (expression in % of non-occluded: eNOS 49.4 ± 11.8% and iNOS 54.5 ± 7.9% vs. non-occluded at 12 h after occlusion; *p *< 0.03). An artificial rise (induced by a peripheral vasodilatation) of the initially decreased collateral blood flow back to pre-occlusion levels reduced collateral macrophage recruitment (macrophages per collateral section: post- 42.5 ± 4.4 vs. artificial pre-occlusion 27.8 ± 2.0; *p *< 0.05) and diminished collateral proliferation (proliferative index: post- 0.54 ± 0.02 vs. artificial pre-occlusion 0.19 ± 0.04; *p *< 0.001) significantly 72 h after femoral artery occlusion.

**Conclusions:**

We propose the following resolution of the "fluid shear stress/NO/macrophage" paradox: Collateral blood flow and NOS expression are initially reduced during arteriogenesis allowing macrophages to accumulate and therewith enhancing collateral proliferation. After homing of macrophages (24 h after occlusion), collateral blood flow and NOS expression recover in order to join the effects of macrophages for restoring blood flow.

## Background

Investigations conducted during the last decade have demonstrated that growth of collateral arteries involves pre-existing arteriolar anastomoses [1]. This phenomenon is restricted to certain vessels within a region and occurs largely outside ischemic territories [2]. These characteristics are distinguished from angiogenesis, the sprouting of capillaries within ischemic territories, and vasculogenesis, that is, the in situ growth of vascular structures from immature precursor cells [3]. The term arteriogenesis has therefore been established for the remodeling process of pre-existing arteriolar shunts into large conductance vessels. It is now widely accepted that flow dependent forces (e.g. fluid shear stress, defined as the viscous drag of flowing blood on the endothelial lining) and macrophage accumulation play a pivotal role during that process [4]. Below, we describe these major components of arteriogenesis.

### Shear stress

As compared to circumferential wall stress, fluid shear stress (FSS) is a rather weak force and mostly acts on endothelial cells. After arterial occlusion, a steep pressure gradient develops between proximal and distal areas of conductance vessels. According to Ohm's law, the increase in proximal to distal pressure gap could only be explained by an increase of flow velocities in pre-existing shunts if resistances and diameters remained the same. Elevated collateral blood flow directly results in elevated collateral fluid shear stress [4,5]. Artificial elevations of collateral flow using arterio-venous shunts lead to vascular remodeling processes very similar to collateral growth [6].

### Monocytes/Macrophages

Early macrophage recruitment during maximal proliferation is another hallmark of collateral artery growth and was first described in dog heart collateral vessels [7]. Subsequent investigations in the rabbit and rat hind limb revealed that macrophage recruitment occurred very early during collateral growth and paralleled maximal proliferation of endothelial and smooth muscle cells [1,8]. Several studies demonstrated that enhancement of macrophage recruitment and survival accelerated collateral growth. In this context, the chemokine MCP-1 (monocyte chemotactic protein-1), is a strong chemotactic factor for monocytes/macrophages, and a potent stimulator of arteriogenesis [9]. Animals deficient in the MCP-1 receptor CCR2 (C-C chemokine receptor 2) demonstrated reduced arteriogenic potential [10], underscoring the importance of MCP-1 in arteriogenesis. Macrophages secrete the majority of growth factors during maximal proliferation [9,11].

### Nitric oxide

Even if recent studies provide strong evidence that arteriogenesis requires nitric oxide (NO) [12], its specific role during that process remains elusive.

On one hand, it was demonstrated that increased blood flow leads to higher nitric oxide (NO) production by the endothelium via stimulation of endothelial nitric oxide synthase (eNOS) expression [13] and activation [14]. Indeed, transcripts for eNOS and inducible nitric oxide synthase (iNOS) were found to be up-regulated on both transcriptional and translational levels during arteriogenesis [4]. Furthermore, N_omega_-nitro-L-arginine-methyl-ester (L-NAME), a potent NOS inhibitor, markedly inhibited the effects of increased FSS [15] on arteriogenesis. In addition, NO enhances proliferation and migration of endothelial cells in vitro [16] and transgenic mice lacking the eNOS showed reduced angiogenic and arteriogenic capabilities [17,18].

On the other hand, NO reduces MCP-1 expression in macrophages and endothelial cells [19,20] and leads to decreased expressions of cell adhesion molecules such as CD11, CD18, VCAM-1 (vascular cell adhesion molecule-1), and ICAM-1 (intercellular adhesion molecule-1) [21,22], counteracting an adhesion of monocytes to the activated endothelial lining. Furthermore, macrophage accumulation occurs under low rather than elevated flow [23] challenging the hypothesis that increased FFS promotes arteriogenesis.

### The shear stress/nitric oxide/macrophage paradox

Thus, the effect of increased flow (fluid shear stress) on NO expression and monocyte recruitment in the context of arteriogenesis appears paradoxical: On the one hand, elevated blood flow leads to increased local levels of NO, which has been shown to promote collateral growth in several studies [12,24]. At the same time, increased flow and NO levels impedes monocyte homing and macrophage accumulation, likewise critical steps during collateral growth. For example, macrophages and monocytes accumulate during collateral growth, suggesting an important role during collateral proliferation.

In the present study we provide a possible explanation for this paradox and hypothesize that a very early phase of collateral growth is characterized by low collateral blood flow conditions, allowing macrophages to adhere, and thus, enhance collateral proliferation.

## Methods

### Animal model

A rat model of peripheral artery insufficiency with unilateral femoral artery occlusion was used as described previously [1]. Experiments were conducted on 103 male Spraque Dawley rats weighting 350 - 450 g. All surgical procedures were performed under general anesthesia induced by CO_2_/O_2 _inhalation and continued by intra-peritoneal injections of ketamin (100 mg/kg bodyweight; Atarost GmbH & Co, Twistringen, Germany) and xylazin 2% (5 mg/kg bodyweight; Bayer Vital GmbH, Leverkusen, Germany). Either MCP-1 (450 ng/ml in PBS) or PBS (phosphate-buffered saline as vehicle) was administered directly into the collateral circulation via osmotic mini pumps (2 ml model, 10 μl/h for 7 d, Alzet, Palo Alto, USA). The non-specific NO-synthase inhibitor L-NAME (Sigma-Aldrich, St. Louis, USA) was given through the drinking water, which was available fresh daily beginning 2 d before surgical procedures and continued throughout the whole study (7 d). Each rat received 65-70 mg L-NAME per kg and per day. Rats of the non-L-NAME groups received ordinary tap drinking water. The investigation conforms with the *Guide for the Care and Use of Laboratory Animals *published by the US National Institutes of Health (NIH Publication No. 85-23, revised 1996). The study was performed according to section 8 of the *German Law for the Protection of Animals *and was approved by the local ethic committee (TV No. 5402, Behörde für Umwelt und Gesundheit der Freien Hansestadt Hamburg, 18.09.2002).

### Experiments on the early phase (0 - 72 h after initiation of collateral growth, Figure 1A + B)

**Figure 1:**
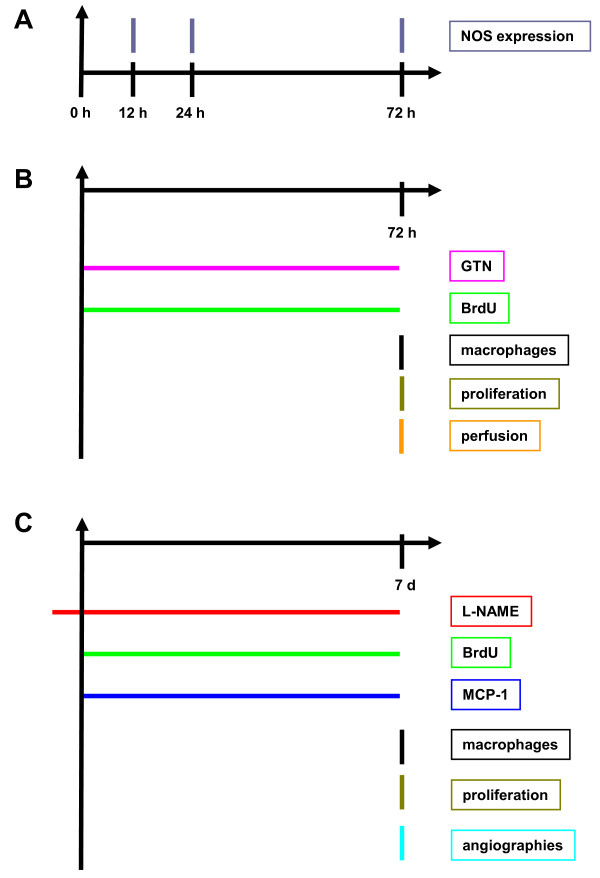
**Experimental set-up**. demonstrated on a post-mortem angiogram (7 d after femoral artery occlusion).

#### Western blot analyses

In order to examine temporal patterns of the expression level of NO producing enzymes in collateral vessel walls we performed quantitative Western blot analyses at three different time points (12 h, 24 h, 72 h) after initiation of arteriogenesis. Fifteen rats were included in these analyses. Main collateral vessels (rudimental ischiadic arteries) from femoral occluded and non-occluded hind limbs were carefully prepared and liberated from all excess tissue including surrounding nerves, veins, and fibrous tissue in cold PBS. The samples were frozen and homogenized in liquid nitrogen. The pulverised tissue was vortexed with ice-cold homogenization buffer and centrifuged at 3000 × *g *for 5 min to remove insoluble material. Sodium dodecylsulfate polyacrylamide gel electrophoresis (SDS-PAGE) was performed in 10% slab gels and transferred electrophoretically onto nitrocellulose membranes using a Bio-Rad Minigel System (Bio-Rad, Hercules, USA). Each lane was then loaded with 20 - 30 μg protein. For immunodetection, unspecific binding sites were then saturated by immersing the membranes for 2 h in the appropriate blocking buffer (in accordance to data sheet). Then, membranes were incubated for another 2 h with the primary monoclonal antibodies [eNOS and iNOS (BD Transduction Laboratories, San Jose, USA) and α-actinin (Sigma-Aldrich, St. Louis, USA)], all applied antibody dilutions, blocking and washing buffers and secondary antibodies were used in accordance to the appropriate data sheets). Membranes were then washed trice in wash buffer and subsequently incubated for 1 h with peroxidase labelled anti-rabbit or anti-mouse IgG antibodies (Vector Labs, Burlingame, USA; dilution 1:10000). Membranes were processed with the enhanced chemiluminescence (ECL) Western blotting detection system (GE Healthcare Europe GmbH, München, Germany). The obtained X-ray-pictures were scanned and evaluated densitometrically by using ScanPack software (Biometra, Göttingen, Germany). All bands were standardized against α-actinin.

#### Collateral blood flow measurements

**Figure 2:**
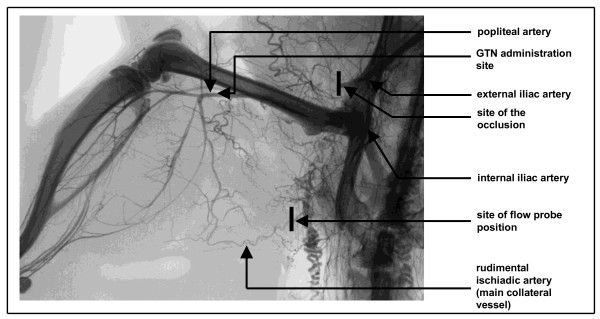
**Experimental set-up**. from short-term (early phase) experiments (A + B) from 0 - 72 h and from mid-term (later phase) experiments (C) 7 d after femoral artery occlusion.

Because NOS expression levels are blood flow/shear force dependent, our NOS expression data needed to be correlated to collateral blood flow changes during arteriogenesis. Eighteen rats were included in these analyses. A perivascular flowprobe (Transonic Systems Inc., Ithaca, USA) was positioned at the stem zone of the remnant of the ischiadic artery which surfaces below the gluteal muscle together with the ischiadic nerve and is easily accessible at this part (Figure 2). As demonstrated previously this vessel becomes the main collateral vessel after femoral artery occlusion in the rat hind limb [1]. Previously published corrosion casts from the rat's hind-limb (see [1]) and post-mortem angiographies (see Figure 2) clearly showed that it possesses only a few side branches until it re-anastomoses with the popliteal artery. Thus, flow measured at the stem of the vessel (which is easily accessible in the gluteal sulcus between the gluteal muscle and the head of the triceps femoris muscle) reflects closely the flow encountered in the mid-zone region of the growing collateral vessel. Using wide beam ultrasonic illumination, the Transonic flowmeter subtracts the downstream from the upstream transit times. The difference in the integrated transit times is a measure of true volume flow and expressed in microliter per minute (μl per min). Blood flow velocity in the main collateral vessel was measured at resting conditions before surgical procedures and directly after femoral artery occlusion. To further investigate the effect of an initially increased flow on collateral growth, we administered the NO donor GTN (glyceryl trinitrate) into the distal stump of the occluded femoral artery to break down the increased peripheral resistance. In order to do so, a third blood flow measurement 3 to 10 min after a peripheral intra-arterial (via a catheter placed in the distal stump of the occluded femoral artery) downstream single-shot application of 500 μg GTN (Nitrolingual infus; Pohl Boskamp, Hohenlockstedt, Germany) took place. Repeated measurements of blood flow at later time points could not be obtained due to surgically induced tissue edema and fibrosis and the inability to replace the Doppler probe.

#### Determination of macrophage accumulation

For the assessment of macrophage accumulation, we used two different antibodies against rat macrophage antigens that have been validated in numerous studies [25,26]. We used ED2 (the rat CD163 cell surface glycoprotein; Serotec, Düsseldorf, Germany) and KiM2R (Bachem, Peninsula, USA) to stain for mature macrophages in rat tissue. Markers for immature macrophages (ED2/CD169 and TRPM-3) have been compared in a recent study by our group and yield similar results [25], and were therefore not applied. We conducted analyses to assess macrophage accumulation, proliferation, and tissue perfusion on the same 10 rats. Cryostat acetone fixed sections (7 μm) were incubated with the primary antibody followed by incubation with peroxidase coupled goat-anti-mouse (Dianova GmbH, Hamburg, Germany). Revelation was performed using amino-ethyl-carbacyl in sodium acetate (0.1 M, pH 4.8). Negative controls were obtained for every staining procedure by omitting the first antibody or by using an isotype control. Controls were negative for all immunohistochemical stainings presented in this study.

#### Determination of proliferative index

Along with the macrophage accumulation, we also investigated collateral proliferation. The same 10 rats from the monocyte accumulation analyses (see above) were included. Proliferation index was assessed using the "5-Bromo-2'-desoxy-uridine Labeling and Detection Kit 2" (Roche Diagnostics GmbH, Mannheim, Germany), a Cy-2 conjugated goat-anti-mouse IgG (Dianova GmbH, Hamburg, Germany) antibody and a 0.001% propidium iodide solution as nuclear staining (Sigma-Aldrich, St. Louis, USA) as described previously [1]. In order to quantify monocytes, pictures were taken from 4 to 5 cryo sections of 3 collateral mid-zone segments. The proliferative index was calculated as number of BrdU (5-Bromo-2'-desoxy-uridine) positive nuclei (green fluorescence) divided by the total number of nuclei (red 7-Amino-actinomycin D fluorescence).

#### Microsphere analyses

To assess collateral-dependent tissue perfusion, fluorescent microsphere analyses were conducted, using the same 10 rats as in the monocyte accumulation analyses (see above). We thus followed a protocol used and evaluated in previous studies [27]. A thoracotomy was performed and ultrasonicated red fluorescent microspheres (15 μm, Molecular Probes, Invitrogen Corporation, Carlsbad, USA) were injected in the left ventricle. Microspheres were counted manually by a blinded observer in 70 μm frozen sections from the soleus muscle of both legs (femoral occluded vs. non-occluded). A total of at least 20 microspheres were counted in each slide. Kidneys served as reference organs proximal to the occlusion site.

### Experiments on the later phase (7 d after initiating of collateral growth, Figure 1C)

We functionally investigated the interdependency of macrophage recruitment and NO availability (experimental set-up from Figure 1C) during collateral growth via a combination of enhanced macrophage recruitment (MCP-1 infusion) and NO antagonism (L-NAME administration). Fifty-nine rats were included in these analyses (31 rats for the macrophage accumulation and proliferation analyses and 28 rats for the angiographic experiments). Either MCP-1 or PBS was administered directly into the collateral circulation via osmotic mini pumps. The non-specific NO-synthase inhibitor L-NAME was given through the drinking water, which was available fresh daily beginning 2 d before surgical procedures. Determination of macrophage accumulation and proliferative index was performed as described above; post-mortem angiographies are described below.

#### Post-mortem angiographies

Post-mortem angiographies were obtained as described previously [1,2]. In brief, 12 g of gelatin (Sigma-Aldrich, St. Louis, USA) was dissolved in 100 ml of heated distilled water under continuous steering. Afterward, 60 g of barium sulfate (Merck KGaA, Darmstadt, Germany) was added. Animals were anesthesized again, anticoagulated, and the aorta was cannulated. Subsequently, animals were bled and immersed in water warmed to 37°C. After rinsing the lower part of the body with saline (37°C, at 80-100 mm-Hg), the contrast medium was infused with a pressure of 150-180 mmHg until filling of the distal femoral stump was observed. The animals were immediately placed on crushed ice in order to allow the contrast medium to harden under continuous pressure. Before obtaining the angiographies, the rats were embedded in gelatin (Sigma-Aldrich, St. Louis, USA) to fix the animal in order to obtain an equilibrated thickness for X-ray penetration. X-ray pictures were taken in a X-ray chamber (Model 43855D, Faxitron X-Ray LLC, Lincolnshire, USA) using single paper wrapped films (X-OMAT MA 13 × 18 cm, Kodak GmbH, Stuttgart, Germany) that were exposed to 30 kV for 6 min. Pictures were taken at two different angles in order to allow stereoscopical analysis of vessel architecture. Identification and counting of collateral arteries were performed by using a stereoscope allowing three dimensional identification of stem, mid-zone, and re-entry regions of the collateral vessels.

#### Statistical analysis

Data are represented as mean ± standard error of mean (SEM). Statistical comparisons between groups were performed by using analysis of variance (ANOVA). Bonferroni corrections were employed for inter-group comparisons. P-values below 0.05 were considered significant.

## Results

### Experiments on the early phase (0 - 72 h after initiation of collateral growth, Figure 1A + B)

**Figure 3:**
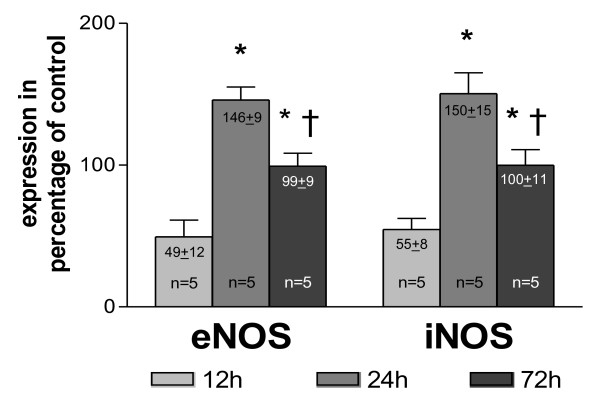
**Quantification of collateral NOS expression**. as percentage of control vessels (main collateral arteries from non-occluded hind limbs) at different time points after femoral artery occlusion, *p < 0.03 vs. 12 h, †*p *< 0.03 vs. 24 h.

To examine the level of NO producing enzymes in collateral vessel walls, quantitative Western blot analyses of NOS expression were performed at different time points after initiation of arteriogenesis (experimental set-up from Figure 1A). As shown in Figure 3, the expression of the NOS was initially (12 h post-occlusion) down-regulated compared to expression levels in non-femoral occluded pre-existing collaterals (*p *< 0.03 vs. non-occluded). 24 h post-occlusion, the expression of both eNOS and iNOS then exceeded the corresponding levels in non-occluded animals by almost 50% (*p *< 0.03 vs. non-occluded). 72 h after occlusion, eNOS and iNOS expression did not differ between occluded and non-occluded animals.

**Figure 4:**
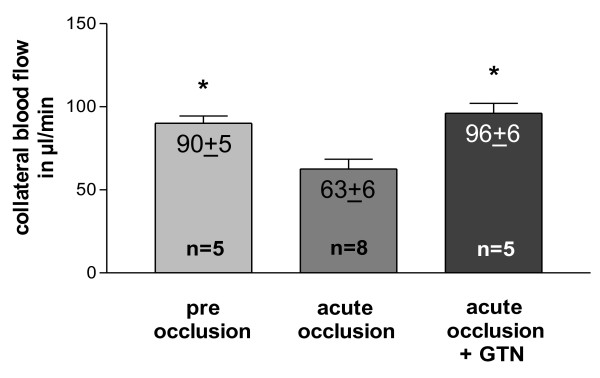
**Quantification of blood flow**. in main collateral artery (rudimental ischiadic artery) at pre and post femoral artery occlusion levels (with or without an acute peripheral intra-arterial administration of 500 μg nitroglycerin [GTN]); *p < 0.01 vs. acute occlusion.

Blood flow in the main collateral vessel was 90.0 ± 4.5 μl/min at resting conditions (Figure 4). After acute femoral artery occlusion, blood flow significantly decreased by almost one-third. An acute peripheral, intra-arterial application of 500 μg GTN into the distal stump of the occluded femoral artery (experimental set-up from Figure 2) restored collateral blood flow to pre-occlusion levels after 3 min.

**Figure 5:**
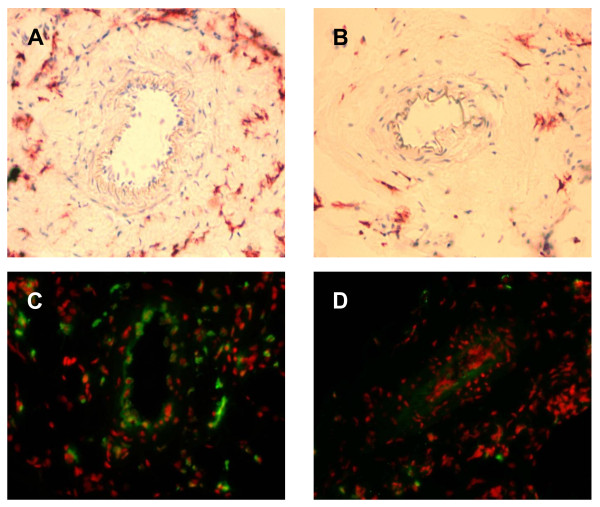
**A + B, Immunohistochemical detection of resident macrophages**. in the absence (panel A; occlusion) and presence (panel B; occlusion + GTN) of continuous, peripheral GTN infusion (10 μg/h over 72 h) 72 h after femoral artery occlusion, 20× magnification. **C + D, Fluorescence immunohistochemical detection of BrdU-incorporated nuclei **(green) in the absence (panel C; occlusion) and presence (panel D; occlusion + GTN) of continuous, peripheral GTN infusion (10 μg/h over 72 h) 72 h after femoral artery occlusion; 20× magnification, 7-Amino-actinomycin D nuclear stain (red fluorescence).

**Figure 6:**
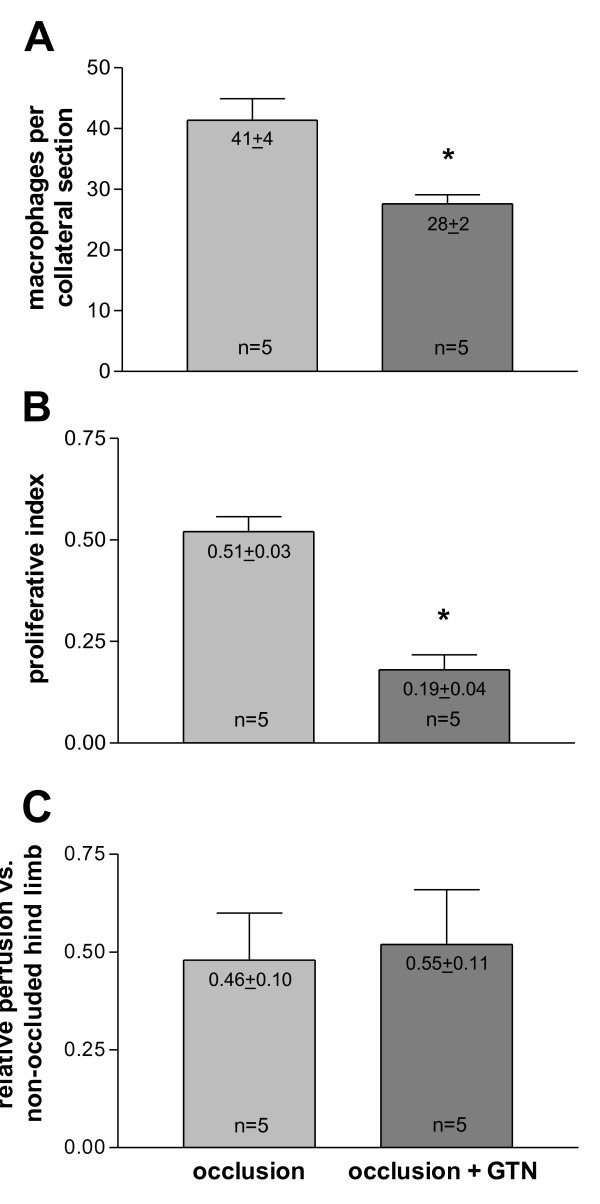
**A, Quantification of resident macrophages**. per collateral section in the absence (occlusion) and presence (occlusion + GTN) of continuous, peripheral GTN infusion (10 μg/h over 72 h) 72 h after femoral artery occlusion, *p < 0.05 vs. occlusion. **B, Quantification of proliferation **in the absence (occlusion) and presence (occlusion + GTN) of continuous, peripheral GTN infusion (10 μg/h over 72 h) 72 h after femoral artery occlusion, *p < 0.001 vs. occlusion. **C, Quantification of collateral-dependent tissue perfusion **in the absence (occlusion) and presence (occlusion + GTN) of continuous, peripheral GTN infusion (10 μg/h over 72 h). Ischemic limb perfusion 72 h after femoral artery occlusion was expressed as a ratio of fluorescent microsphere counts in ischemic soleus muscle and related to non-femoral occluded leg (set as 1).

In order to assess the functional impact of the initial collateral flow depression on macrophage accumulation and collateral proliferation, initial blood flow levels were artificially elevated via infusion of GTN (experimental set-up from Figure 1B). Chronic GTN infusion (over 72 h) significantly reduced the number of pericollateral ED2 positive macrophages by more than one-third (Figure 5A + B and Figure 6A).

This reduced number of macrophages surrounding collateral vessels (72 h after occlusion with ongoing GTN supply) was accompanied by a reduction of collateral proliferation (Figure 5C + D and Figure 6B). Continuous GTN application decreased proliferative index (PI) by almost two-thirds.

To assess collateral-dependent tissue perfusion, fluorescent microsphere analyses were performed. Relative perfusion of ischemic vs. non-ischemic limbs (soleus muscle) 72 h after femoral artery occlusion remained equal in chronic GTN- or non-GTN-treated rats (Figure 6C).

### Experiments on the later phase (7 d after initiating of collateral growth, Figure 1C)

**Figure 7:**
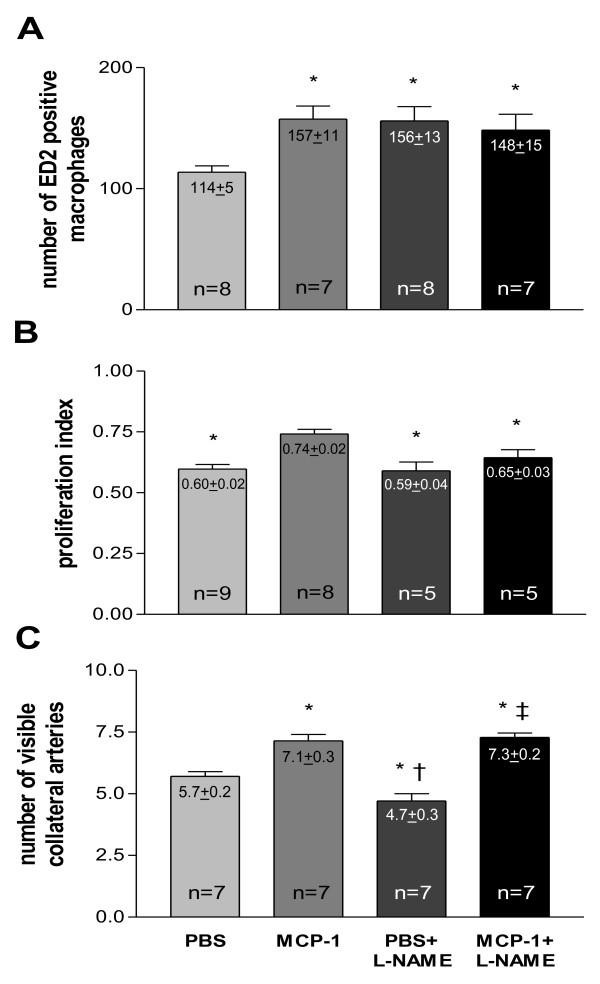
**A, Quantification of resident macrophage**. (mature, ED2 positive) accumulation in vehicle (PBS), MCP-1, vehicle (PBS) + L-NAME and MCP-1 + L-NAME treated animals 7 d after femoral artery occlusion, macrophage score per collateral section, *p < 0.05 vs. PBS. **B, Quantification of collateral proliferation **in vehicle (PBS), MCP-1, vehicle (PBS) + L-NAME and MCP-1 + L-NAME treated animals 7 d after femoral artery occlusion, proliferative index, *p < 0.05 vs. MCP-1. **C, Quantification of angiographically visible collateral vessels **in vehicle (PBS), MCP-1, vehicle (PBS) + L-NAME and MCP-1 + L-NAME treated animals 7 d after femoral artery occlusion, number of visible collaterals, *p < 0.03 vs. PBS, †*p *< 0.001 vs. MCP-1, ‡*p *< 0.001 vs. PBS + L-NAME.

Chronic MCP-1 treatment led to a higher macrophage accumulation around main collateral vessels as compared to the vehicle (phosphate-buffered saline, PBS, treated) group (Figure 7A). Likewise, chronic L-NAME treatment enhanced the number of pericollateral macrophages in the PBS as well as in the MCP-1 group. Consistently, MCP-1 significantly increased proliferation in collateral vessels (Figure 7B). However, NO antagonism (L-NAME) had no effect on collateral proliferation neither in PBS nor in MCP-1 treated animals. Chronic oral L-NAME treatment led to decreased scores of angiographically visible collateral vessels 7 d after femoral artery occlusion (Figure 7C), whereas chronic MCP-1 administration significantly enhanced collateral growth reflected in a larger amount of vessels in the collateral network. This positive effect of MCP-1 could not be antagonized by L-NAME treatment.

## Discussion

The cardinal paradox of the arteriogenesis hypothesis on the initiation of collateral growth was that monocyte accumulation, a crucial step in this process, occurred primarily under low rather than high blood flow conditions [22,23]. However, collateral blood flow/FSS was thought to be elevated during the entire build-up of collateral vessels. Thus far, experimental data on the regulation of blood flow in collateral vessels was missing. In this study, we present a possible solution to this "FSS/NO/macrophage" paradox during collateral growth.

Our study demonstrates that collateral growth is a complex process characterised by a sequence of distinct steps, beginning with an early phase between 0 and 12 h after initiation. This phase is characterized by low collateral flow conditions (Figure 4) and a rather low local NO concentration (Figure 3), allowing macrophages to adhere to the endothelium and to migrate through the collateral vessel wall. A second phase between 12 and 24 h after initiation is characterized by elevated collateral flow and increased NO availability (Figure 3). Collateral proliferation might be set off during this phase. Once migrated, macrophages need a certain degree of NO concentration and elevated flow during this stage of arteriogenesis to differentiate and proliferate locally (Figure 7B).

Our analyses demonstrated that the expression of NOS was significantly down-regulated 12 h post-occlusion (Figure 3), correlating with initially increased monocyte/macrophage recruitment [22,23], but rose significantly beyond expression levels in non-occluded control animals within 24 to 72 h post-occlusion. Based upon these initial findings, we hypothesize that collateral flow and local NO production were initially decreased in collateral arteries, allowing the recruitment of circulating cells, which can differentiate locally during the following days. To assess this hypothesis, we measured blood flow directly in the stem region of the main collateral artery in the rat hind limb (rudimental ischiadic artery) via a transonic flow probe. In agreement with our hypothesis, flow velocities decreased acutely after femoral artery occlusion. Interestingly, a previous study published by our group provided a potential explanation for this observation [2]. In a rabbit model we observed a marked increase in peripheral resistance directly after femoral artery occlusion. This increase in peripheral resistance could explain why the flow remains rather low, despite an increase of the pressure gradient across the collateral circulation.

An acute GTN (a strong vasodilator reducing peripheral resistance) infusion blunted this initial decrease of flow in the collateral vessel (Figure 4). An ongoing infusion of GTN during the first 3 d after femoral artery occlusion (time period of maximal macrophage accumulation) decreased macrophage accumulation and collateral proliferation (Figure 6A + B). These findings indeed support the notion that the initial decrease of flow in collateral vessels is important for collateral macrophage accumulation and, at the same time, for collateral proliferation.

During the later phase of collateral growth, local activation of initially immigrated cells might either be triggered by paracrine mechanisms of the FSS activated endothelium or via tensile forces. These hypotheses are supported by the observation that most proliferation of cells occurs in the direct vicinity of collateral vessels [1]. Because the number of pericollateral macrophages and collateral proliferation was decreased in the "occlusion + GTN" (Figure 6A + B) as compared to "occlusion without GTN" group, we also expected to find a reduced collateral-dependant perfusion. However, finding no substantial difference in tissue perfusion between both groups might be explained by the following: Firstly, the collateral-dependant peripheral tissue perfusion (soleus muscle) is, in fact, reduced due to less pericollateral macrophages, and thus, less proliferation of the upstream lying collaterals, but the effect is abolished by a GTN-induced peripheral vasodilatation. Actually, the fact that collateral-dependant perfusion is not reduced could be an indirect hint that GTN was effective for 72 h and that nitrate tolerance was less likely to have occurred. Secondly, the effect of an enhanced tissue perfusion in the "occlusion" group (due to enhanced collateral proliferation) only occurs on physical activity (when maximal collateral flow is demanded) and not, as in our case, under resting conditions.

After our initial experiments, revealing reduced collateral blood flow and NO availability 12 h after femoral artery occlusion, but increased flow and NO availability thereafter, we administered L-NAME in order to inhibit this early collateral NO peak (occurring 24 h after occlusion). The late phase (chronic) experiments were conducted to investigate the mid- or long-term effects of the now modulated early phase. In order to do so, we assessed the impact of chronic NOS inhibition (oral L-NAME administration) and macrophage chemotaxis (intracollateral MCP-1 administration) on macrophage accumulation and collateral proliferation as well as on angiographically visible collaterals.

MCP-1 alone led to a late (7 d after femoral artery occlusion; Figure 7A) enhanced number of pericollateral macrophages and therewith to an increased collateral proliferation (Figure 7B) and a larger amount of visible collaterals (Figure 7C). These results are in line with previous publications [1,8,9]. L-NAME alone as well as in combination with MCP-1 led to a late enhanced macrophage accumulation (7 d after occlusion; Figure 7A) possibly by further reducing already decreased early NO bioavailability (12 h after occlusion; Figure 3).

Surprisingly, the L-NAME group did not show an increased collateral proliferation, despite the increased number of macrophages as compared to the PBS group. It appears that if L-NAME inhibits the collateral NO peak (occurring 24 h after occlusion; Figure 3) macrophages are not able to enhance collateral proliferation (not even in the presence of MCP-1). One could speculate that NO is essential for the differentiation and local proliferation of already migrated macrophages. The fact that the total number of collaterals was reduced (Figure 7C) in the L-NAME group is most likely due to a vasoconstrictive effect of L-NAME and in line with previous reports by Mees at al. showing that the transient decrease in collateral blood flow in eNOS knockout animals is mainly due to the inability to vasodilate [24]. Furthermore, it appears that MCP-1 somehow abolished the vasoconstrictive effect of L-NAME (reflected in an increased number of collaterals; Figure 7C). This finding is rather unexpected because a direct vasodilatory effect of MCP-1 has not, to our knowledge, been described yet.

Our study highlights that collateral growth is a multi-phase process with different distinct stages. Elevated CBF/FSS and increased NO production appear to be restricted to later phases of collateral growth. Thus, studies investigating the impact of an artificially elevated FSS on collateral growth, for instance via construction of an arterio-venous shunt, are capable of elucidating certain mechanisms of collateral growth but will possibly miss others. In this context, it is noteworthy that in the first study investigating the effect of shunting on collateral growth, shunts were created 7 d after femoral artery occlusion [6]. According to our findings, the short period of low CBF/FSS and macrophage recruitment had long passed at this time point. Furthermore, several studies showed that the peak of macrophage recruitment and proliferation occurred during the first week after femoral artery occlusion and was followed by a prolonged phase of vascular remodeling [1,2,28]. Thus, the arterio-venous shunt in the study of Pipp et al. probably enhanced and prolonged the remodeling phase of collateral growth. At this time point, neither macrophage recruitment nor proliferation is very prominent anymore [2,8,28]. The later extensive remodeling phase of collateral growth rather appears to be driven by migratory processes and possible further stimulation of progenitor cells that are mainly vascular resident at this time [25,29]. While a recent study failed to show an effect of the small GTPase Rac2 on collateral growth [30], Eitenmuller et al. were able to demonstrate that the Rho-GTPase pathway was activated during arterio-venous shunting of collateral vessels at the time of femoral artery occlusion [15]. This pathway plays an important role in migratory processes. L-NAME abolished the positive effect of arterio-venous shunting on collateral conductance. Unfortunately, neither macrophage accumulation nor collateral proliferation was investigated in this study. Taken together, the findings of Eitenmuller et al. and Pipp et al., in conjunction with our data, suggest that arterio-venous shunting enhances collateral conductance via prolongation and augmentation of the remodeling phase of collateral growth that occurs after the initial phase characterized by vascular proliferation and macrophage accumulation [1,28]. As the results of our study do not contradict investigations showing a dramatically increasing remodeling process of collateral vessels after construction of an arterio-venous shunt, they also do not contradict findings demonstrating the importance of the NO system in stem cell mobilization during vascular growth, because our study focuses on local kinetics in collateral vessels and not on the bone marrow niche that may be subject to quite different kinetics at the same time [31]. Finally, the question arises whether our model of an acute occlusion of the femoral artery resembles the pathophysiological situation encountered in human patients. In the human heart, this certainly only pertains to the situation of an acute myocardial infarction. In this setting, the kinetics of collateral growth are indeed similar to the ones seen in our model [32].

### Limitations of study

The major limitation of our study is that we provide data only on CBF and not on FSS (the main stimulus of collateral growth). Collateral shear stress can be calculated by using the following equation assuming Newtonian fluid dynamics, if the collateral blood flow *(Q)*, the blood viscosity *(η)*, and the internal radius of the collateral vessel *(R) *are provided.

$$FSS=\frac{4\times \eta \times Q}{\pi \times R^{3}}$$

The equation demonstrates that increased blood flow will only directly result in increased FSS if blood viscosity and the radius/diameter of the vessel remained the same.

Our experimental set-up (with extended surgery) only allowed us to measure temporal changes in collateral blood flow velocities but not changes in collateral diameter in the same animal at the same time. Obtaining the collateral diameter is rather challenging as the main collateral vessel is rather small (according to our data, around 150 to 200 μm [1]) and it also must be obtained very accurately because, according to the Hagen-Poiseuille law, a 10% change in radius alters FSS by approximately 30% and a 20% change equates to 70%. Although collateral diameters can be obtained (e.g. with the use of MR angiographies), it was not feasible in our study after all the applied surgical techniques. The level of NOS expression can give a rather indirect hint to how shear stress changes during arteriogenesis since NOS expression is FSS dependent.

It furthermore might seem rather puzzling that we measured NOS expression on one hand (Figure 1A) and administered an NO donor on the other hand (Figure 1B). It is important to note that, as shown in Figure 1, NOS expression measurements and administration of NO donors (GTN) were performed in independent experiments; specifically, NOS expression therefore was never measured in the presence of an NO donor (GTN). GTN was administered in order to reduce peripheral resistance and to open up the ischemic microcirculation, but certainly has several limitations. However, alternative substances such as selective resistance vessel vasodilators did not seem to be suitable in our experimental setting. For example, adenosine has a short life span and had not, to our knowledge, been used in osmotic pumps. Even if the GTN was given peripheral to the collateral network (Figure 2) in a small dose, a low degree of reflux into the collateral network cannot be excluded. NO generated from GTN could also directly affect collateral monocyte/macrophage adherence and cell proliferation after entering the systemic circulation and the collateral network. Nitrate tolerance normally develops after 72 h of continuous high-dose exposure, while we used low-dose administration up to a maximum of 72 h.

Because we focused on macrophages only, we could have missed effects of other immune and inflammatory cells on collateral proliferation. There is, for example, strong evidence that CD4-positive lymphocytes provide an arteriogenic effect by recruiting macrophages to the site of active collateral growth [33].

## Conclusions

We present, for the first time, direct evidence of natural blood flow kinetics in collateral vessels after femoral artery occlusion. According to our data, CBF as well as NOS expression is initially reduced during arteriogenesis, allowing macrophages to accumulate and therewith enhancing collateral proliferation. These results help clarifying important questions concerning the arteriogenesis hypothesis, in particular, the interplay of CBF/FSS, NO, and macrophage recruitment.

## Competing interests

The authors declare that they have no competing interests.

## Authors' contributions

HBS, RM, KR, and WDI carried out the experimental work and drafted the manuscript. WDI, JW, WL, and HS conceived the study, participated in its design and coordination, and helped to draft the manuscript. All authors read and approved the final manuscript.
